# Metallothionein and neurodegenerative diseases

**DOI:** 10.4103/NRR.NRR-D-25-00011

**Published:** 2025-08-13

**Authors:** Yufeng Cheng, Yujia Zhao, Ce Chen, Feng Zhang

**Affiliations:** 1 Key Laboratory of Basic Pharmacology of Ministry of Education and Joint International Research Laboratory of Ethnomedicine of Ministry of Education and Key Laboratory of Basic Pharmacology of Guizhou Province and Laboratory Animal Center, Zunyi Medical University, Zunyi, Guizhou Province, China; 2 Sino-Jan Joint Lab of Natural Health Products Research, School of Traditional Chinese Pharmacy, China Pharmaceutical University, Nanjing, Jiangsu Province, China

**Keywords:** Alzheimer’s disease, amyotrophic lateral sclerosis, Huntington’s disease, metal chelator, metal homeostasis, metal ion, metallothionein, neurodegenerative diseases, Parkinson’s disease, Wilson’s disease

## Abstract

Neurodegenerative diseases, which mainly include Alzheimer’s disease, Parkinson’s disease, amyotrophic lateral sclerosis, Wilson’s disease, and Huntington’s disease, are a group of disorders characterized by loss of neurons in the brain and spinal cord. However, the underlying pathogenetic mechanisms of these disorders remain unclear. The metal ion hypothesis is considered a possible cause of a variety of neurodegenerative diseases. This hypothesis posits that the homeostatic imbalance of metal ions leads to oxidative stress, neuroinflammation, excessive aggregation of pathological proteins, and other serious consequences in neurons. The powerful endogenous metal ion chelator metallothionein plays an important role in regulating metal ion homeostasis to alleviate neurodegenerative diseases. This article provides an overview of the pathogenesis of neurodegenerative diseases in relation to metal ions such as copper, iron, and zinc and the contribution of metallothionein to the regulation of metal ion homeostasis. The review focuses on the role of metal ions in the course of neurodegenerative diseases and the molecular mechanisms through which endogenous metallothionein ameliorates metal ion overload to alleviate neurodegenerative diseases. A thorough understanding of these molecular mechanisms can provide a theoretical foundation for the development of new therapeutic strategies, with the aim of more effectively treating these devastating diseases in the future.

## Introduction

Neurodegenerative diseases are central nervous system (CNS) disorders presenting with progressive degeneration and necrosis of neurons, and are generally characterized by the accumulation of misfolded proteins and mitochondrial damage (Soto and Pritzkow, 2018). Neurodegenerative diseases mainly include Alzheimer’s disease (AD), Parkinson’s disease (PD), amyotrophic lateral sclerosis (ALS), Wilson’s disease (WD), and Huntington’s disease (HD) (Chi et al., 2018). Different diseases affect distinct areas of the brain and show unique pathological features and clinical symptoms (**Additional Table 1**).

**Additional Table 1:** Characterization of Alzheimer's disease, Parkinson's disease, amyotrophic lateral sclerosis, Wilson's disease, and Huntington's disease

	Alzheimer's disease	Parkinson's disease	Amyotrophic lateral sclerosis	Wilson's disease	Huntington's disease
Pathological features	Aβ-protein aggregation; neurofibrillary tangle deposition (Puzzo et al., 2015)	α-Synuclein aggregation; loss of dopamine neurons (Tysnes and Storstein, 2017)	Cu/Zn superoxide dismutase mutations (Tokuda et al., 2014); aggregation of TAR DNA-binding protein 43 in the cytoplasm (Liao et al., 2022)	Kayser-Fleischer ring (Hegde and Kumar, 2023)	Mutant huntingtin protein aggregation (Walker, 2007)
Pathogenesis	Metal toxicity (Chen et al., 2023); neuroinflammation (Barage and Sonawane, 2015)	Intestinal flora dysbiosis; metal toxicity; mitochondrial dysfunction; neuroinflammation; oxidative stress	Excitatory amino acid toxicity; genetics; metal toxicity; neurotrophic factor deficiency; oxidative stress	Mutation in the ATPase Cu transporting beta gene leading to Cu metabolism disorders (Hahn, 2014)	Mutation in the Huntington's disease gene
Clinical symptoms	Aphasia (Ozben and Ozben, 2019); behavioral disorders; cognitive dysfunction	Bradykinesia; muscle rigidity (Collier et al., 2017); postural instability; resting tremor	Cone beam damage; dysphagia (van Es et al., 2017); myasthenia gravis; myofascial tremor; medullary palsy	Extrapyramidal injury	Involuntary choreiform movements; mental disturbances; progressive dementia
Therapeutic measures	Antiinflammatory drugs; antioxidant drugs; calcium antagonists; cholinesterase inhibitors; drugs that inhibit Aβ formation and deposition; free radical scavengers; glutamate receptor antagonists	Aducanumab (Dhillon, 2021; Budd Haeberlein et al., 2022); amantadine; catechol-o- methyltransferase inhibitors; dopamine agonists; levodopa; monoamine oxidase inhibitors	Gene therapy (Storkebaum et al., 2005); neural stem cell therapy (Mazzini et al., 2015)	Penicillamine (Lucena- Valera et al., 2023); Zn supplementation (Yang, 2007)	Gene therapy; immunotherapy (Jamwal et al., 2020); medication therapy (Salman et al., 2022); neural stem cell therapy (Yoon et al., 2020)

Aβ: Amyloid-β; Cu: copper; Zn: zinc.

AD is a progressive and irreversible neurodegenerative disease. The pathological features of AD include the aggregation of amyloid-β (Aβ) peptides and deposition of hyperphosphorylated tau protein in neurofibrillary tangles in the brain (Puzzo et al., 2015). The pathogenesis of AD is characterized by the activation of the neuroinflammatory response due to the presence of insoluble Aβ peptides (Barage and Sonawane, 2015), the hyperphosphorylation of tau proteins that disrupts abnormal cellular functions (Bloom, 2014), and the combination of metal ions with Aβ peptides to enhance the toxicity of the peptides (Chen et al., 2023). The predominant clinical symptoms of AD are cognitive dysfunction, behavioral disorders, and aphasia (Ozben and Ozben, 2019). The existing pharmacological interventions for AD are mainly categorized as cholinesterase inhibitors, glutamate receptor antagonists, calcium channel antagonists, free radical scavengers, antioxidant agents, and anti-inflammatory drugs. Notably, Aducanumab, a human IgG1 monoclonal antibody, shows targeted binding to Aβ soluble oligomers and insoluble protofibrils. The U.S. Food and Drug Administration approved the use of this drug for the treatment of AD-derived mild cognitive impairment, but the launch of Aducanumab is still controversial (Dhillon, 2021; Budd Haeberlein et al., 2022).

PD is the second-most common neurodegenerative disease in the world, occurring mainly in older individuals over 65 years of age (Li et al., 2024). The main pathological features of PD are the loss of dopamine (DA) neurons in the substantia nigra and the aggregation of α-synuclein to form Lewy bodies (Tysnes and Storstein, 2017). The clinical symptoms of PD are bradykinesia, postural instability, resting tremor, and muscle rigidity (Collier et al., 2017). In addition, most patients also exhibit non-motor symptoms such as sleep disorders, depression, gastrointestinal dysfunction, and constipation (Kalia and Lang, 2015). The main pathogenic mechanisms underlying PD include abnormal aggregation of α-synuclein, oxidative stress, mitochondrial dysfunction, neuroinflammation, homeostatic imbalance of metal ions, and dysbiosis of intestinal flora (Sampson et al., 2016; Jankovic and Tan, 2020). Clinical treatment of PD is predominantly pharmacological, focusing on the induction of DA synthesis by DA neurons, agonizing DA receptors, or blocking the degradation of DA to compensate for the loss of DA (Jankovic, 2019). In detail, commonly used drugs are levodopa, DA agonists, monoamine oxidase inhibitors, amantadine, and catechol-o-methyltransferase inhibitors.

ALS is a fatal neurodegenerative disease that causes degeneration of motor neurons in the brainstem and spinal cord (Rué et al., 2019). Depletion of TAR DNA-binding protein 43 (TDP-43) in the nucleus and aggregation of TDP-43 in the cytoplasm are pathologic markers of ALS (Liao et al., 2022). Disruption of intracellular copper homeostasis is a common pathogenic factor for different superoxide dismutase-1 (SOD-1) mutants, which can cause familial ALS (Tokuda et al., 2014). Several hypotheses have been proposed to explain the pathogenesis of ALS, including genetic mechanisms, oxidative stress, excitatory amino acid toxicity, neurotrophic factor deficiencies, and disruptions in metal homeostasis. Clinically, ALS patients initially present with symptoms such as myasthenia gravis, myofascial tremor, medullary palsy, and cone beam damage, which then progress to dysphagia and respiratory muscle weakness (van Es et al., 2017). Although interventions such as gene therapy (Storkebaum et al., 2005) and neural stem cell therapy (Mazzini et al., 2015) have been employed to treat ALS, these methods cannot completely cure ALS.

WD, also known as hepatolenticular degeneration, is a chromosomally recessive disorder characterized by a mutation in the ATPase copper transporting beta (*ATP7B*) gene, which results in impaired copper metabolism (Hahn, 2014). It is identified on the basis of changes in the basal ganglia on magnetic resonance imaging of the brain (Kitzberger et al., 2005). Moreover, at least 50% of patients with WD show Kayser-Fleischer rings, which is the easiest method for diagnosis of WD (Hegde and Kumar, 2023). The pathogenesis of WD is attributable to ATP7B protein dysfunction resulting from mutations in the *ATP7B* gene, impairment of copper excretion from the biliary tract, and copper binding to ceruloplasmin, all of which lead to accumulation of copper in the liver (Pfeiffer, 2011). The main clinical manifestation of WD is neurologic injury, particularly in the extrapyramidal system. The primary objective of drug therapy is chelating excess copper. For example, penicillamine, the first-choice drug for WD, chelates copper in the body and promotes copper excretion (Lucena-Valera et al., 2023). Moreover, zinc supplementation is considered to be an effective adjuvant and can promote urinary copper excretion in patients with WD (Yang, 2007).

HD is a neurodegenerative disorder featuring motor dysfunction, behavioral/psychiatric symptoms, and cognitive dysfunction (D’Egidio et al., 2024). Migration of the Huntington gene on autosome 4 triggers the formation of mutated protein inclusion bodies, which cluster together and accumulate in massive molecular clusters in the brain, interfering with neuronal cell function (Walker, 2007). Clinically, the disease is characterized by a triad of involuntary choreiform movements, mental disturbances, and progressive dementia. A combination of medication (Salman et al., 2022), neural stem cell therapy (Yoon et al., 2020), gene therapy, and immunotherapy (Jamwal et al., 2020) was incidentally shown to optimize the pathology of HD, although no definitive therapies are available owing to the absence of specific therapeutic targets for the disease.

A common pathological feature of neurodegenerative diseases is the excessive aggregation of pathological proteins, with metal ions being one of the significant factors affecting protein aggregation. These metal ions can interact with proteins to alter the rate of protein aggregation. However, this dependency is not always a direct relationship. Under normal physiological conditions, protein aggregation typically does not occur, but it can be induced in the presence of certain metal ions. The extent of the effect of metal ions on protein aggregation is not yet fully understood, since this effect depends on a variety of factors, such as the pH value, the concentration of the aggregating proteins, and the concentration levels of metal ions (Poulson et al., 2019). Over the past few years, a growing number of studies have confirmed that essential metal ions (copper, iron, and zinc) are indispensable for vital functions and play important roles in the maintenance of brain functions (Mezzaroba et al., 2019). Metal ion disorders may cause permanent damage to the brain, leading to neurodegenerative diseases.

Thus, maintaining a dynamic balance of essential metal ions is crucial. Metal chelators play an important role in neurodegenerative diseases by forming stable complexes with metal ions, which helps control the concentration and toxicity of metal ions (Gucký and Hamuľaková, 2024). MT, a key protein involved in metal ion homeostasis in mammals, can chelate metal ions through metal-thiolate bonds with cysteine (Cys) residues (Samson and Gedamu, 1998).

This review begins with the origins, classification, structure, and biological functions of MT. It then proceeds to discuss the influence of metal ions on neurodegenerative diseases. Finally, the review predicts how the presence of MT can regulate the homeostasis of metal ions to improve the progression of neurodegenerative diseases and provides a summary of the current therapeutic effects of metal-chelating agents as well as the prospects of this molecular mechanism in future clinical therapies.

## Search Strategy

The literature cited in this narrative review was published between 1957 and 2025 and was identified by searching the PubMed database. We used the following combination of keywords to initially select the articles to be evaluated: “Metallothionein,” “Metallothionein and neurodegenerative diseases,” “Metal chelator,” “Metallothionein and Alzheimer’s disease,” “Metallothionein and Parkinson’s disease,” “Metallothionein and amyotrophic lateral sclerosis,” “Metallothionein and Wilson’s disease,” and “Metallothionein and Huntington’s disease.” We included review and research articles covering MTs and their relationship with neurodegenerative diseases such as AD, PD, ALS, WD, and HD; research published between the discovery of MT and 2025; and studies available through the PubMed database. The search strategy and screening criteria adopted these specific keywords and its combinations. These criteria were intended to ensure that the most relevant and scientifically valuable studies were enrolled to provide a better overview of the role of MT in neurodegenerative diseases.

## Research History of Metallothionein

**Figure 1:**
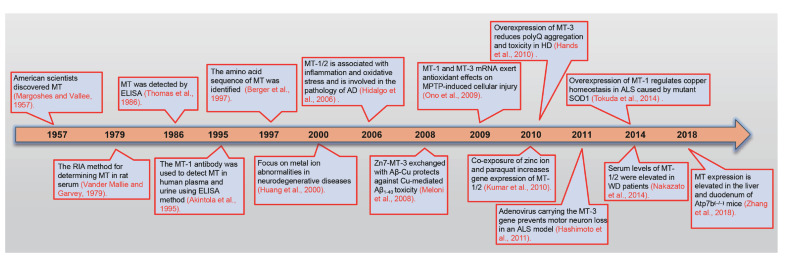
Development of MT and neurodegenerative diseases. From the discovery of MT in 1957 to its research in ALS models in 2018, the research progress on MT in diseases such as AD, HD, ALS, and WD has been summarized. Key events include the discovery of MT-3 in the brain, the role of MT-1 and MT-3 in antioxidant stress, the effect of MT-3 on polyQ aggregation, and the regulation of copper homeostasis by MT in ALS. Created with Servier Medical Art (https://smart.servier.com/), licensed under a Creative Commons Attribution 4.0 Unported License. AD: Alzheimer’s disease; ALS: amyotrophic lateral sclerosis; ELISA: enzyme-linked immunosorbent assay; MT: metallothionein; PolyQ: polyglutamine; RIA: radioimmunoassay; SOD1: superoxide dismutase 1; WD: Wilson’s disease.

MT was first identified in horse renal cortex in 1957 (Margoshes and Vallee, 1957). Subsequently, detection methods for MT and its functions in neurodegenerative diseases were further investigated (**Figure 1**). Vander Mallie and Garvey (1979) established the radioimmunoassay method for determination of MT in rat serum. Thomas et al. (1986) developed an enzyme-linked immunosorbent assay for detection of MT, which was not significantly different from the radioimmunoassay method. Akintola et al. (1995) developed an enzyme-linked immunosorbent assay with detection limits up to 5 ng/mL for the determination of MT in human plasma and urine. In 1997, over 170 MTs were identified and their amino acid sequences were determined, and MT-3 was characterized as a brain-specific member that shows biological functions different from other MTs (Vašák and Meloni, 2017). In 2000, a study began to focus on the abnormalities of metal ions in neurodegenerative diseases, such as the aggregation of zinc ions within the brain in AD, but did not focus directly on MT (Huang et al., 2000). Hidalgo et al. (2006) suggested that MT-1/2 and MT-3 played specific roles in brain physiology and that MT-1/2 may be involved in the pathology of AD, being closely related to inflammation and oxidative stress responses. By 2008, Meloni et al. (2008) proposed that zinc-complexed MT-3 (Zn7-MT-3) could prevent copper-mediated Aβ_1–40_ toxicity through metal exchange with Aβ-Cu. Ono et al. (2009) found that induction of MT-1 and MT-3 mRNA expression produced antioxidant effects on 1-methyl-4-phenyl-1,2,3,6-tetrahydropyridine-induced cellular damage. Kumar et al. (2010) further observed that co-exposure of zinc ions and paraquat increased gene expression of MT-1/2. Hands et al. (2010) found that overexpression of MT-3 significantly reduced polyglutamine (polyQ) aggregation and toxicity. For ALS, Hashimoto et al. (2011) demonstrated that adenoviruses carrying the *MT-3* gene prevented motor neuron loss in ALS model mice and prolonged lifespan, providing new potential targets and ideas for the treatment of ALS. Nakazato et al. (2014) found that MT-1 overexpression regulated copper homeostasis and ameliorated the pathological features of ALS caused by mutant SOD1. Moreover, Nakazato et al. (2014) found that serum MT-1/2 levels were significantly elevated in WD. Zhang et al. (2018) further identified elevated MT expression in the liver and duodenum of Atp7b^–/–^ mice, which may be an adaptive response of the body to aberrant copper metabolism to help regulate copper homeostasis and alleviate copper toxicity.

## Metallothionein

### Origins and definition of metallothionein

In 1957, while studying the biological role of metals, the American scientist Margoshes first isolated cadmium-binding protein from the cortex of horse kidney. Since this protein had a low molecular weight and high sulfhydryl content and could bind heavy metal ions in large quantities, it was named MT (Margoshes and Vallee, 1957). MT can be triggered by various stimuli, including metals, cytokines, cytotoxic drugs, and oxidative stress (Haq et al., 2003). The molecular structure of MT contains a large number of Cys residues, which account for almost 30% of its total amino acid composition. These Cys residues form metal ion-binding sites to facilitate the formation of metal-mercapto clusters (Asselman et al., 2012). In 1985, the International Committee on Metallothionein Nomenclature defined MT on the basis of the following characteristics: (1) low molecular weight, approximately 6000–7000 Da, (2) high metal ion content, generally combining seven metal ions, (3) high Cys content, free of aromatic amino acids and histidine, and (4) enriched with Cys-X-Cys, Cys-X-Cys-Cys and Cys-X-X-Cys sequences (X was a non-Cys amino acid) (Rauser, 1990).

### Classification of metallothionein

MT is present in various mammals and abundantly enriched in different tissues, such as liver, eye, and CNS. MT and its analogs are also found in higher plants and prokaryotic and eukaryotic microorganisms. The complexity of MT genes determines the complexity of MT spatial structure. Human MT is generally categorized into four classes: MT-1, MT-2, MT-3, and MT-4 encoded by 11 genes located in the chromosome 16 cluster. In particular, MT-1 and MT-2 are present in most tissues of the body, with the highest levels observed in visceral tissues (liver and kidney). Furthermore, MT-1 and MT-2 are confirmed to confer neuroprotective functions (Liu et al., 2020). MT-3 is expressed in the CNS in mammals, and it plays an important role in the programmed death of tissues and brain cells (Vašák and Meloni, 2017). MT-4 is mainly observed in squamous epithelial cells, where it was indispensable for the regulation of zinc or copper metabolism (Meloni et al., 2006). Interestingly, MT-1 and MT-2 are located in the spinal cord and brain, specifically in astrocytes, whereas MT-3 is found in neurons (Juárez-Rebollar et al., 2017).

### Structure of metallothionein

MT without bound metal ions is called a thioprotein. The structure of the protein is chain-like and unstable, and it folds into an ordered structure after binding metal ions (Romero-Isart and Vasák, 2002). MT has two independent domains: the C-terminal α domain, which encompasses amino acids 31–68 and can bind four divalent metal ions, and the N-terminal β domain, which consists of amino acids 1–30 and can bind three divalent metal ions (Nath et al., 1988). The intermediate region, which consists of amino acids 29–31, can serve as a linkage, yielding a molecule with a dumbbell-shaped conformation. This specialized structure enables MT to show excellent thermal stability (Vergani et al., 2005).

### Physiological functions of metallothionein

#### Metal detoxification effects

The ability to chelate metal ions shown by MT has been primarily attributed to the high abundance of Cys residues with sulfhydryl groups, and it represents one of the most crucial pathways for metal detoxification in living cells (Vijver et al., 2004). MT is induced by metallic cadmium in spermatogonia, spermatocytes, and Sertoli’s cells derived from testicular tissue, which, in turn, alleviates cadmium-induced tissue damage (Kusakabe et al., 2008). Zinc supplementation can antagonize cadmium-induced apoptosis, reactive oxygen species (ROS) production, and mitochondrial depolarization in macrophages by promoting MT expression (Zhang et al., 2019b). In vanadium metal–affected mussels, a significant positive correlation was observed between MT and vanadium concentration (Amiard et al., 2008).

#### Elimination of reactive oxygen species

Heavy metals produce a huge number of free radicals in the organism, causing lipid peroxidation of cellular unsaturated fatty acids, destroying cell membranes, and leading to abnormal metabolism in the organism (Rochette et al., 2022). MT is currently recognized as the most powerful antioxidant, demonstrating significant efficacy in scavenging a diverse array of free radicals, including hydroxyl and superoxide radicals. Its reaction rate constant with hydroxyl radicals is approximately 300 times that of glutathione (Miyazaki and Asanuma, 2023). Furthermore, in comparison with glutathione, MT shows approximately 50-fold higher antioxidant activity in mitigating oxidative DNA damage and approximately 10-fold higher activity in preventing lipid peroxidation (Bensellam et al., 2021). The nucleophilic nature of the sulfhydryl groups of MT and the kinetic instability of its metal atoms allows easy interaction with electrophilic substances, such as free radicals. For example, human MT-3 has been shown to prevent ferric ion-nitrilotriacetic acid and hydroperoxide-induced DNA damage (You et al., 2002). In atherosclerotic lesions, MT is expressed only in smooth muscle cells and participates in protecting against cell-produced ROS (Göbel et al., 2000). Furthermore, MT overexpression can prevent diabetes-induced cardiomyocyte defects by inhibiting excess ROS production (Ye et al., 2003).

#### Physiological functions

Under physiological conditions, MT is the metabolic regulation center of essential trace elements in the body, and it is involved in the storage, transport, and metabolism of trace elements (Yang et al., 2024). In addition, MT influences the synthesis of hormones, including thyroid hormones, glucocorticoids, and growth hormones, and regulates the stability and biological activity of hormones.

In conclusion, MT plays an important role in regulating metal ion homeostasis due to its special structure and diversified biological functions. Consequently, MT shows wide application potential in neurodegenerative diseases affected by metal ions.

## Metallothionein Expression and Induction in Neurodegenerative Diseases

The promoter regions of the genes encoding MT-1 and MT-2 contain multiple elements that regulate their expression, including the TATA box, CpG box, and basal level element, as well as several specific regulatory sequences, such as the metal-responsive element, antioxidant-responsive element, glucocorticoid-responsive element, and thyroxine-responsive element (Andrews, 1990; Ogushi et al., 2020). Under stress conditions, such as exposure to metals, hormones, hypoxia, or inflammation, various factors bind to these transcriptional regulatory structures and response elements within the MT gene promoter, enhancing MT synthesis at the transcriptional level (Raudenska et al., 2014; Takahashi, 2015). However, certain proteins, such as PZ120, which contains a zinc finger structural sequence, can bind to the transcriptional start site of the MT-2A gene promoter, thereby inhibiting MT-2A expression (Tang et al., 1999). Although the MT-3 gene promoter also contains a metal-responsive element, its expression does not appear to be induced by metals such as zinc, suggesting that the regulation of MT-3 expression differs from those of MT-1 and MT-2 (Bousleiman et al., 2017).

MT-1 and MT-2 expressions are synchronously regulated, and their synthesis can be induced by a variety of factors, including ROS, metal ions, oxidative stress, glucocorticoids, and cytokines (Ghoshal and Jacob, 2001). These inducers are present in several regions of the CNS, such as the cerebral cortex, brainstem, hippocampus, thalamus, basal forebrain, spinal cord, neocortex, olfactory bulb, cranial nuclei, and cerebellum (Penkowa, 2006; Santos et al., 2012). MTs are mainly expressed in the cytoplasm, mitochondria, and lysosomes, as well as in numerous cells such as astrocytes, meningeal cells, ventricular cells, choroid, and the arachnoid membrane (Nishimura et al., 1992; Suzuki et al., 1994). Interestingly, MT-1 and MT-2 are present both intracellularly and extracellularly. Moreover, neurons can internalize these MTs into the cytoplasm via megalin, a member of the low-density lipoprotein receptor family (Pedersen et al., 2009). Under normal conditions, microglia, oligodendrocytes, and neuronal cells do not typically express MT-1 or MT-2; however, these proteins may be induced in response to brain injury (Agullo et al., 1998; Leung et al., 2018). In contrast, MT-3 is predominantly expressed in the CNS, especially in neurons (Yu et al., 2001). MT-3 is more abundantly expressed in the cerebral cortex, hippocampus, amygdala, and nuclei of the basal cerebellum (Masters et al., 1994). A major point of distinction between MT-3 and MT-1/2 is their response to stress conditions: while MT-1/2 expression is upregulated under such circumstances, MT-3 is not activated. MT-3 is also not regarded as inducible due to its lower metal affinity than MT-2, and its gene transcription is not stimulated by metals (Jacob et al., 1999; Vasák and Hasler, 2000; Palumaa et al., 2005).

Several heavy metals, including lead, mercury, cadmium, copper, and zinc, have been shown to induce the synthesis of MT. Upon entering the CNS, these heavy metals bind to MTs, thereby mitigating the toxic effects of the metals. For example, mice subjected to chronic exposure to a volcanic environment showed a marked increase in the expression of MT-2A in regions such as the hippocampus, ventricular choroid plexus epithelial cells, blood vessels, white matter, and Purkinje cells. This upregulation was attributed to the persistent accumulation of heavy metals in the environment, potentially representing a detoxification mechanism employed by the organism to counteract heavy metal toxicity (Navarro-Sempere et al., 2023). Furthermore, inflammatory processes may significantly contribute to the induction of MT expression, and for neurodegenerative diseases such as multiple sclerosis (MS), the inflammatory response induces the expression of MT-1/2, which serves a neuroprotective function (Penkowa et al., 2003). Moreover, the bacterial endotoxin lipopolysaccharide as well as inflammatory cytokines such as interleukin-1 (IL-1), IL-6, and tumor necrosis factor-α (TNF-α) have been shown to contribute to increased expression levels of MTs in the brain (Hernández and Hidalgo, 1998).

3xTg-AD mice show increased Aβ deposition and exacerbated neuronal damage, and MT-3 overexpression improved cognitive functions in these mice (Lv et al., 2024). MT overexpression reduced dopamine neuron apoptosis in 1-methyl-4-phenyl-1,2,3,6-tetrahydropyridine- or 6-hydroxydopamine (6-OHDA)-induced PD models (Ebadi et al., 1998; Wanpen et al., 2004). MT-1 overexpression prolonged survival and slowed motor function degeneration in SOD1^G93A^ transgenic mice (Tokuda et al., 2014). High-level expression of MT-1/2 reduced copper toxicity in the liver and brain in ATP7B-deficient mice (WD model) (Meacham et al., 2018). However, the existing studies are mostly based on animal models, and the specific roles of MTs in human diseases require further validation, especially since the subtypes (MT-1/2/3/4) may show differences in functions.

## Metal Ions and Neurodegenerative Diseases

Metal ions, such as copper, iron, and zinc, are involved in a variety of physiological activities in the CNS, including neurotransmitter transmission, redox balance, and cell metabolism (Zhang et al., 2023). Homeostatic imbalances of these metal ions have been linked to the development of neurodegenerative diseases such as AD, PD, ALS, WD, and HD. Metal ions (copper, iron, and zinc) play key roles in the CNS, and the maintenance of their homeostasis is essential for normal nerve function. (1) Metal ion entry and excretion pathways in CNS: Iron entry into the CNS through the blood–brain barrier (BBB) relies primarily on transferrin receptor-mediated endocytosis (Zheng and Monnot, 2012; Galy et al., 2024). Copper is transported from the blood to the brain through the BBB, where endothelial cells of BBB facilitate copper takeup from the blood via copper transporter-1 (CTR1) and release it into the brain parenchyma via ATPase copper transporting alpha (ATP7A) (Scheiber et al., 2010). Iron is released into the brain interstitial fluid via FPN1 at the abluminal membrane of brain microvascular endothelial cells and then transported via transferrin receptor 1-mediated endocytosis to enter the medial side of the BBB and ultimately the peripheral circulation (Mills et al., 2010; Chen et al., 2025). Zinc enters and leaves the CNS primarily through families of zinc transporters such as the Zrt/Irt-like protein (ZIP) and zinc transporter (ZnT). The ZIP family (SLC39A) is responsible for transporting zinc ions from the extracellular or intracellular compartment to the cytoplasm, while the ZnT family (SLC30A) is responsible for transporting zinc ions from the cytoplasm to the extracellular compartment or the organelle. These transporters are crucial for maintaining zinc homeostasis within the CNS (Gower-Winter and Levenson, 2012; Kambe et al., 2014; McAllister and Dyck, 2017). (2) Basic physiological functions of copper, iron, and zinc: Iron is involved in mitochondrial energy transduction, enzyme catalysis, myelin formation, and neurotransmitter synthesis and catabolism. Iron is also involved in oxidative phosphorylation and NO metabolism as a cofactor (Ward and Cloonan, 2019; Yiannikourides and Latunde-Dada, 2019; Dietz et al., 2021; Khattar et al., 2021; Chen et al., 2025). Copper acts as a cofactor for a number of enzymes involved in cellular respiration, free radical detoxification, iron metabolism, and synthesis of neurotransmitters, neuropeptides, and hormones (Tapiero et al., 2003; Ruiz et al., 2021). Zinc is involved in intercellular signaling as a neurotransmitter, regulating cellular signal transduction and synaptic plasticity. Zinc is also involved in the regulation of γ-aminobutyric acid synthesis, which affects neuronal excitability (Zhang et al., 2022; Chen et al., 2024). (3) The mechanisms underlying abnormal aggregation: Abnormal accumulation of metal ions such as copper, iron, and zinc in the CNS may be related to dysfunction of the respective metal transporter proteins, metabolic dysregulation, and interactions with specific proteins (e.g., α-synuclein, tau proteins), which, in combination with these mechanisms, can lead to abnormal accumulation of metal ions in the CNS and thereby trigger neurodegenerative disorders.

### Metal ions and Alzheimer’s disease

#### Copper and Alzheimer’s disease

The human body contains approximately 100 mg of copper, of which approximately 75% is present in the skeleton and muscle tissue, with the liver, brain, blood, heart, and kidney accounting for most of the remaining copper (Burkhead and Collins, 2022). In biological systems, copper ions exist in Cu^+^ (reduced) and Cu^2+^ (oxidized) forms that undergo interconversion through redox reactions (Kim et al., 2008). The correlation between copper levels and AD has not been fully clarified. Copper ion homeostatic imbalance is a common phenomenon in the pathogenesis of AD. Moreover, copper levels have been shown to vary across different regions of the brain in patients with AD, and copper mis-localization has been shown to affect neuronal functions (Mao et al., 2012; Rembach et al., 2013). Additionally, metal ions can affect Aβ aggregation (Liu et al., 1999). A study has shown that copper levels in Aβ plaques are apparently increased in patients with AD than in the brains of normal age-matched individuals (Mot et al., 2011). However, copper levels were lower in tissues that surrounded Aβ (Zheng et al., 2010).

Copper is also involved in cellular oxidative stress and Aβ production. Cu^+^ promotes hydroxyl radical production by engaging in the Fenton and Haber-Weiss reactions. The amyloid precursor protein (APP) contains two copper-binding sites, which are located in the C-terminal region within the Aβ peptide and the N-terminal region. When Cu^2+^ binds to the N-terminal copper-binding site, it can be reversed to form Cu^+^, after which Cu^+^ enters the cell via the high-affinity CTR1, promoting the formation of free radicals and causing neurotoxicity (Logeman et al., 2017; Mathys and White, 2017). Furthermore, copper can trigger the phosphorylation and aggregation of tau protein, enhancing the neurotoxicity of tau aggregates (Voss et al., 2014). In addition, copper has been shown to activate nuclear factor-κB signaling in microglia, increasing the release of inflammatory factors such as IL-1β, IL-6, and TNF-α to damage neurons. Copper has been also shown to downregulate the expression of low-density lipoprotein-associated receptor protein-1, affecting the ability to scavenge Aβ (Kitazawa et al., 2016). In summary, copper plays an important role in the pathogenesis of AD, which involves the disruption of copper homeostasis, interactions with Aβ and tau proteins, destruction of glial cell function, and neuroinflammation. These findings provide new research directions for the treatment and prevention of AD.

#### Iron and Alzheimer’s disease

The brain tissue of patients with AD shows iron accumulation, which leads to lipid peroxidation and downregulation of glutathione peroxidase 4 (GPX4) to trigger the activation of ferroptosis signaling and thereby cause neuronal damage (Reichert et al., 2020; Chen et al., 2021). Meanwhile, the utilization of a ferroptosis inhibitor has been shown to attenuate the neuronal death and memory impairment induced by Aβ aggregation, further demonstrating that ferroptosis drives AD progression (Bao et al., 2021). Five genes, including *JUN*, *SLC2A1*, *TFRC*, *ALB*, and *NFE2L2*, have been shown to be closely associated with ferroptosis in patients with AD and could effectively distinguish AD patients from controls (Wang et al., 2022c). These findings indicate a close relationship between iron levels and AD.

Ferroportin 1 (FPN1) is the only cellular iron export protein in mammals (Yanatori et al., 2016). The secreted form of APP (sAPP) on the neuronal cell surface stabilizes FPN1 function and plays an important role in iron export. In AD, the neuronal non-amyloid pathway is inhibited, and the resultant decrease in sAPP production causes suppression of FPN1 activity. Inhibition of neuronal iron output has been shown to cause iron accumulation (Ashraf and So, 2020; Wang et al., 2022a). Simultaneously, the reduced expression of FPN1 in AD leads to elevated sub-iron pools in cells, which generate hydroxyl radicals via the Fenton reaction and further produce ROS to induce ferroptosis and promote AD progression (Bao et al., 2021). In addition, one of the characteristics of AD is reduction in GPX4 levels, which is consistent with the features of ferroptosis. GPX4 is considered a key regulator of ferroptosis, since it inhibits lipid peroxidation by directly reducing membrane lipid hydroperoxides to lipid alcohols (Yang and Nao, 2023). Moreover, the iron-responsive element has been found in the 5′-untranslated region of APP mRNA. Iron can act at the iron-responsive element site of APP mRNA, thereby enhancing the translation and expression of endogenous APP (Zhou and Tan, 2017). In autopsies of patients with AD, misfolding of tau proteins was confirmed to cause iron accumulation (Spotorno et al., 2020). Tau protein deposition promoted the upregulation of heme oxygenase-1 expression. Then, heme oxygenase-1 catalyzed more Fe^2+^ to potentiate oxidative stress and trigger neuronal lipid peroxidation (Schipper, 2011). Interestingly, high levels of iron have been shown to co-localize with tau proteins. Iron not only triggered the activity of multiple kinases that affected the hyperphosphorylation of tau proteins, but also induced aggregation of hyperphosphorylated tau proteins and increased the formation of neurofibrillary tangles (Rao and Adlard, 2018).

#### Zinc and Alzheimer’s disease

Excess zinc in the brain has been linked to the pathology of AD. Obvious Zn^2+^ presence has been reported in the neuronal bodies and dendrites in Aβ plaques and neurofibrillary tangles (Suh et al., 2000). Aβ possesses a metal-binding domain that zinc can bind to and induce the aggregation of Aβ (Kulikova et al., 2015). Surprisingly, low levels of zinc also contribute to the development of AD. Lack of zinc weakens SOD activity. Consequently, the Zn-SOD system is not easily formed, and its inability to scavenge free radicals in the body can lead to cerebral vascular damage and subsequent dementia (Marchetti, 2014).

Zinc overload can enhance Aβ deposition by increasing APP expression, thereby damaging spatial learning and memory in mice (Wang et al., 2010a). Zn^2+^ promotes tau protein phosphorylation by inhibiting protein phosphatase 2A activity and induces tau protein aggregation by triggering tau protein cross-linking (Sun et al., 2012). Likewise, excess zinc directly binds to tau proteins and enhances their aggregation (Wang et al., 2020). Interestingly, zinc released from presynaptic neurons and astrocytes has been shown to activate NADPH oxidase, increase neuronal ROS production and inflammatory responses in microglia, and ultimately trigger apoptosis (Wang et al., 2020).

### Metal ions and Parkinson’s disease

#### Copper and Parkinson’s disease

Under physiological conditions, copper catalyzes the formation of ROS through Fenton chemical reactions (Wang et al., 2012). In the CNS, the areas of the brain that contain the highest amount of copper are the basal ganglia, hippocampus, and cerebellum (Madsen and Gitlin, 2007). Copper homeostasis is regulated by copper transporters such as CTR1 and ATP7A/ATP7B. Dysfunction of these copper transporters leads to increased levels of free copper ions. Subsequently, copper catalyzes the generation of highly reactive hydroxyl radicals from hydrogen peroxide via the Haber-Weiss cycle, causing oxidative damage to DA neurons (Giampietro et al., 2018). Cities with higher industrial emissions of copper or manganese have been reported to show a higher incidence of PD, implying that environmental metal exposure may be a risk factor for PD (Willis et al., 2010). In addition, in a 6-OHDA-induced PD rat model, analysis using inductively coupled plasma mass spectrometry showed that copper content in the substantia nigra region increased with time after 6-OHDA treatment (Tarohda et al., 2005). However, 6-OHDA does not fully cause the pathological features of PD since it does not induce the formation of Lewy bodies, a key pathological marker of PD. In contrast, PD models induced by 1-methyl-4-phenyl-1,2,3,6-tetrahydropyridine or transgenic methods may more accurately reflect the pathological features and symptoms of PD. In the 1-methyl-4-phenyl-1,2,3,6-tetrahydropyridine-induced PD mouse model, increased copper levels enhanced α-synuclein metabolism and accumulation, causing oxidative stress and dopaminergic neuron death (Lan et al., 2016). Thus, copper overload may be a factor influencing the pathogenesis of PD.

Moreover, in the substantia nigra of patients with PD, the CTR1 protein level is significantly reduced. CTR1 is a copper transporter, and changes in its levels may affect the homeostasis of copper ions. Meanwhile, in activated astrocytes, the expression of MT subtypes, such as MT1E, MT1F, MT1G, MT1H, MT1M, MT1X, and MT2A, is obviously increased. These findings suggest that these MT subtypes may be involved in responding to changes in copper ions during PD pathology (Michael et al., 2011). High concentrations of copper have also been detected in both the cerebrospinal fluid and substantia nigra of PD patients, and copper shows a high affinity for α-synuclein, which accelerates the aggregation of α-synuclein (Wang et al., 2010b; Abbaoui et al., 2017). Additional studies have also provided direct evidence of α-synuclein aggregation by copper ions (Binolfi et al., 2006; Li et al., 2020). Using atomic force microscopy and molecular dynamics to simulate copper ions, α-synuclein, an individually insoluble filamentous structure, was found to progressively aggregate to form a dense network of protofibrils upon exposure to copper (Synhaivska et al., 2022). Together, these findings reveal the potential roles of copper ion metabolism disorders and MT expression changes in the pathogenesis of PD. They also clarify how copper ions may exacerbate neurodegeneration by promoting the aggregation of α-synuclein.

#### Iron and Parkinson’s disease

Epidemiologic investigations have indicated that an environment enriched in iron is associated with a higher incidence risk of PD (Powers et al., 2009). Furthermore, iron levels in the substantia nigra region of the brain in patients with PD have been shown to be higher than those in normal individuals (Jiang et al., 2017). Correspondingly, early dyskinesia was found to be alleviated by the clinical utilization of deferiprone, an iron chelator, to reduce iron levels in PD patients (Devos et al., 2014). In addition, patients with PD show abnormal expression of most proteins in the iron metabolism pathway, such as transferrin receptor 1 and divalent metal transporter 1, affecting the homeostasis of iron ions in the brain (Lee et al., 2010; Boag et al., 2022). Collectively, homeostatic dysregulation of iron ions can be considered to be closely associated with the pathogenesis of PD.

The formation of Lewy bodies by α-synuclein aggregation is known to be a pathological marker of PD. α-Synuclein contains binding sites for iron ions, which trigger the overexpression of α-synuclein when DA neurons are exposed to high iron concentrations. Meanwhile, iron ions cause the formation of α-synuclein protein aggregates and fibrosomes, inducing conformational changes of α-synuclein and damaging DA neurons (Chen et al., 2019a). In particular, ROS produced by α-synuclein and lipid peroxides in the cell membrane may trigger ferroptosis, which is accompanied by a reduction in GPX4 levels. The use of iron chelators can protect neurons (Wu et al., 2018; Ding et al., 2023). In addition, iron can directly interact with α-synuclein in Lewy bodies, which were degraded mainly via the autophagic lysosomal pathway. Impairment of this pathway can lead to massive deposition of ferritin and further cause autophagy disorders in DA neurons, resulting in increasing levels of α-synuclein and ROS to induce DA neuronal damage (Chen et al., 2019b). Under conditions involving abundant deposition of iron ions, the hydroxyl radicals generated by Fenton’s chemical reaction would promote abnormal activation of microglia, releasing massive amounts of pro-inflammatory factors to damage neurons (Song et al., 2018). In addition, at the genetic level, iron ions inactivate the Parkin gene, an E3 ubiquitin ligase, in DA neurons, which inhibit the degradation of α-synuclein, leading to its aggregation and subsequent mitochondrial dysfunction to induce DA neuronal death (Ganguly et al., 2020).

#### Zinc and Parkinson’s disease

The role of zinc ion levels in PD has been a topic of debate. On one hand, patients with PD have been reported to show low blood and serum levels of zinc ions (Ahmed and Santosh, 2010; Brewer et al., 2010). On the other hand, elevated levels of zinc ions have been identified in the serum and cerebrospinal fluid of patients with PD (Squitti et al., 2007; Hozumi et al., 2011).

These variations in zinc ion levels may imply that patient populations with different characteristics, such as sample size, age distribution, and disease severity, show differences in zinc ion levels. Furthermore, PD is a progressive disease, and zinc ion levels may show different changes at different stages of the disease. Evaluation of dynamic changes requires detailed time-series analysis to better understand the role of zinc ions in the progression of PD. In a 1-methyl-4-phenyl-1,2,3,6-tetrahydropyridine-induced PD mouse model, the increased levels of cytoplasmic zinc ions implied degeneration of DA neurons (Lee et al., 2009). Similarly, a 6-OHDA-induced PD rat model showed increased levels of zinc and copper in all regions on the dopaminergic pathway (Tarohda et al., 2005). Moreover, massive release of the excitatory neurotransmitter glutamate was observed in an PD animal model, which subsequently led to homeostatic dysregulation of Zn^2+^ and mitochondrial dysfunction in DA neurons (Iovino et al., 2020; Campanelli et al., 2022).

Like iron, zinc can influence the progression of PD by inducing the aggregation of α-synuclein. Through analysis of the binding sites of zinc ions and α-synuclein by nuclear magnetic resonance spectroscopy, the interaction sites of zinc ions and α-synuclein protein were identified to be around the Asp121, Asn122, and Glu123 amino acid residues (Valiente-Gabioud et al., 2012). In addition, zinc also affected the ubiquitin protease system, which impaired the degradation of α-synuclein to cause DA neuronal damage (Kumar et al., 2018). Since oxidative stress was particularly prominent in the pathogenesis of PD, it induced the release of zinc ions from metalloproteins and increased the concentration of zinc ions in the brain, thereby activating neuronal apoptosis (Aizenman et al., 2000).

### Metal ions and amyotrophic lateral sclerosis

#### Copper and amyotrophic lateral sclerosis

In ALS, copper-zinc SOD1 is a copper and zinc ion complexing metalloprotein that plays a crucial role in converting high ROS and superoxide levels into oxygen molecules and hydrogen peroxide (Sanghai and Tranmer, 2021). A mutation in the *SOD1* gene has been identified as the main cause of familial ALS disease (Rosen, 1993; Tafuri et al., 2015). Copper levels are increased in the cerebrospinal fluid of ALS patients and mice. However, lower copper levels have been observed in SOD1 mutant mice (Lelie et al., 2011; Roos et al., 2013).

In some ALS patients, the SOD1 gene was mutated, and misfolded SOD1 may accumulate in the motor neurons of the spinal cord (Anzai et al., 2020). In addition, an imbalance in copper ion homeostasis is a common feature in SOD1 mutants because it alters the copper transport system, causing copper accumulation in the spinal cord of mice (Tokuda et al., 2013). Copper chaperone proteins can transport copper to Cu/Zn-SOD and activate its chaperone protein SOD1 to produce antioxidant effects (Leitch et al., 2009). During ALS, copper chaperone proteins interact incorrectly with mutant SOD1, leading to the accumulation of unstable SOD1, which tended to become pro-oxidant and thus exerted toxic effects on motor neurons (Gil-Bea et al., 2017). Interestingly, in another study, in mice expressing SOD1 mutants, including G93R, G37R, and G85R, the absence of copper chaperone proteins resulted in a significant reduction of SOD1. However, the onset and progression of the disease were unchanged (Subramaniam et al., 2002).

#### Iron and amyotrophic lateral sclerosis

In patients with ALS and multiple mouse models, GPX4-mediated anti-iron association defense was shown to be impaired in the CNS. Overexpression of human GPX4 in SOD1 mutant G93A mice, an animal model of ALS, reversed ferroptosis and ameliorated lipid peroxidation and motor neuronal death (Wang et al., 2022b). Co-administration of the iron chelators VAR10303 and high-calorie/energy-supplemented diet cured SOD1^G93A^ mice, improved their motor behavior, prolonged survival time, and reduced iron accumulation and motor neuron deficits in the spinal cord (Golko-Perez et al., 2017). Moreover, patients with sporadic ALS showed elevated lipid peroxidation in the cerebrospinal fluid and plasma and decreased levels of reductive glutathione in the motor cortex, indicating the occurrence of ferroptosis (Simpson et al., 2004).

#### Zinc and amyotrophic lateral sclerosis

Multiple studies have reported higher levels of zinc in the cerebrospinal fluid of ALS patients than those in age-matched control participants (Kanias and Kapaki, 1997; Hozumi et al., 2011). Moreover, the accumulation of large amounts of zinc in the spinal cord of SOD1^G93A^ transgenic mice has been shown to result in the degeneration of numerous spinal motor neurons and the activation of astrocytes. In addition, elevated zinc levels have been shown to induce lipid peroxidation, damaging Zn^2+^ homeostasis by triggering the release of zinc from MT (Kim et al., 2009). A pathological feature of ALS is the presence of ubiquitinated neuronal aggregates containing TDP-43. Expression of endogenous TDP-43 by zinc-treated neuronal cells induced depletion of TDP-43 expression and the formation of TDP-43-positive aggregates. Surprisingly, this particular interaction was independent of copper and iron (Caragounis et al., 2010).

### Metal ions and Wilson’s disease

#### Copper and Wilson’s disease

Copper is a cofactor for enzymes in key metabolic pathways. Mutations in copper metabolism genes and copper metabolism disorders can result in diseases from either copper excess or copper deficiency (Scheiber et al., 2013). WD is a typical example of copper overload. Patients with WD have higher hepatic copper levels than healthy people (Ala et al., 2007). In these patients, the inability to excrete copper in the bile causes copper accumulation in the liver, which can subsequently extend to other organs, including the brain. Moreover, copper deposits may also be observed in the cornea in patients with WD, a phenomenon known as Kayser-Fleischer rings (Członkowska et al., 2018). In addition, high levels of copper were detected in almost all brain regions of patients with WD (Dusek et al., 2015).

Copper-induced mitochondrial dysfunction may be associated with WD. The hepatocytes of ATP7B knockout rats showed remarkable accumulation of mitochondrial copper, with concentrations reaching twice that of controls at 30–50 days of age and escalating to 10 times the control level by 90 days (Zischka et al., 2011). In addition, the levels of inflammatory factors (IL-1β, IL-18, IL-6, and TNF-α) were elevated in the brains of WD mouse models, and the underlying mechanisms may be related to an overload of copper-mediated activation of NOD-like receptor family pyrin domain containing 3 (NLRP3) inflammasome in microglia (Dong et al., 2021).

#### Iron and Wilson’s disease

WD is a typical liver disease characterized by copper accumulation. Interestingly, patients with WD may develop secondary hemochromatosis, and the underlying pathologic mechanisms are related to ceruloplasmin, which was an important bridge for hepatic copper and iron biochemistry (Collins et al., 2010). Ceruloplasmin is responsible for copper transport and plays a critical role in copper mobilization and secretion in addition to its ferroxidase activity (Shiono et al., 2001; Collins et al., 2010). Fe^2+^ can undergo de-electronization to form Fe^3+^ in the presence of iron ferroxidase (Doguer et al., 2018). However, iron transporter proteins only load Fe^3+^, not Fe^2+^. Thus, in WD, the deficiency in ceruloplasmin leads to impaired oxidation and transport of iron and increased iron storage, ultimately causing iron accumulation in the liver and other organs.

#### Zinc and Wilson’s disease

The copper toxicity in WD mainly manifests in the liver and brain. To date, therapeutic interventions have focused on the development of anti-copper drugs. The four main anti-copper drugs available at present are tetrathiomolybdate, zinc, tretinoin, and penicillamine (Schilsky et al., 2022). Of these, zinc is primarily used as the first-line drug for maintenance treatment, particularly in non-symptomatic patients. Its mechanism of action involves inducing intestinal mucosal cells to generate MT, which binds copper in intestinal mucosal cells that are excreted through the feces, thereby reducing the intestinal absorption of copper.

### Metal ions and Huntington’s disease

#### Copper and Huntington’s disease

In the postmortem frozen brain tissue of patients with HD, copper content was determined by inductively coupled plasma spectroscopy, and increased copper concentration was detected in the putamen of the striatum (Dexter et al., 1991). Copper accumulation promoted the aggregation of Huntingtin (HTT) proteins, and copper directly interacted with histidine residues at the N-terminus of the proteins (Fox et al., 2007). In addition, the first 171 amino acids of the wild-type HTT protein and its glutamine-expanded mutant form interacted with copper, but not iron. Moreover, the use of copper chelators inhibited HTT aggregation, whereas copper supplementation increased the formation of aggregates (Fox et al., 2007). Copper may promote the progression of HD by inactivating mitochondrial dehydrogenases and decreasing the production of NADH (Pamp et al., 2005; Lautrup et al., 2019).

#### Iron and Huntington’s disease

In patients with HD, elevated iron levels were seen mainly in the striatum and pallidum (Bartzokis et al., 1999). In the early stages of HD, iron ion levels increase with age, suggesting that iron plays an important role in the progression of HD. In addition, in the brains of transgenic HD mice, increased levels of free radicals were generated due to the increased Fe levels and elevated levels of oxidative stress–related damage (Dexter et al., 1991). Meanwhile, the increased oxidative stress stimulation led to the aggregation of mutant HTT proteins and proteasome dysfunction. However, the use of the chelator desferrioxamine increased the expression of HTT proteins, indicating that HTT proteins were indispensable for iron homeostasis (Hilditch-Maguire et al., 2000).

#### Zinc and Huntington’s disease

The onset of early symptoms of HD was previously demonstrated to be associated with synaptic transmission dysfunction, and mutant HTT was markedly toxic to synaptic vesicles and synaptic transmission functions (Smith et al., 2005; Steinert et al., 2012). During synaptic activity, free zinc ions within the synaptic vesicles were released into the synaptic gap to modulate multiple postsynaptic receptor functions, and free zinc was almost specifically expressed in the synaptic vesicles of zincergic neurons (a subtype of glutamatergic neurons) (Frederickson and Bush, 2001). At present, reports describing the role of zinc ions in the pathogenesis of HD are limited. Interestingly, in the HD model using *Caenorhabditis elegans*, when the nematode was exposed to zinc for a long time, it showed aggregation of polyQ proteins in neurons, whereas the flavonoid rutin acted as a metal chelator and protected the nematode against zinc ion damage (Cordeiro et al., 2023).

## Metallothionein, Metal Ions, and Neurodegenerative Diseases

On the basis of the information describing the molecular mechanisms of neurodegenerative diseases in the previous sections, this section attempts to address the relationships among MT, metal ions, and neurodegenerative diseases.

### Metallothionein, copper, and neurodegenerative diseases

***Metallothionein, copper, and Alzheimer’s disease***

**Figure 2:**
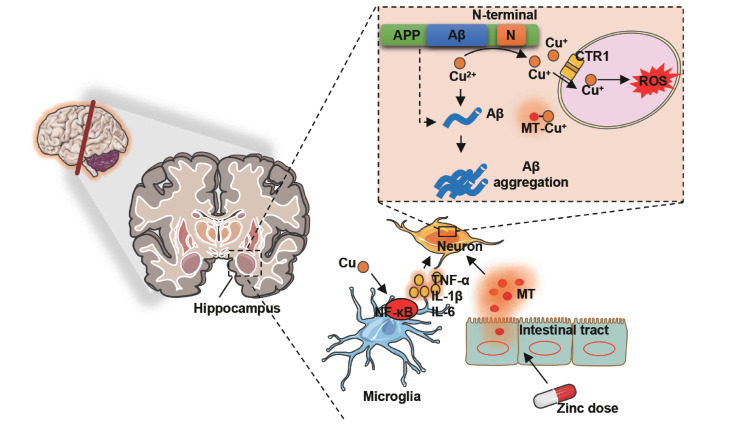
MT, copper, and AD. In AD, excessive accumulation of Cu^2+^ led to the aggregation of Aβ. At the same time, Cu^2+^ interacted with the copper-binding site of the N-terminal residue of APP to become the more toxic Cu^+^, which entered the cell via the action of CTR1 to generate many ROS. In addition, copper ions activate NF-κB signaling in microglia to produce proinflammatory factors, such as TNF-α, IL-1β, and IL-6, that damage neurons. However, zinc administration induced the production of intestinal MT to chelate excess copper ions in neurons to alleviate the disease process of AD. Created with Servier Medical Art (https://smart.servier.com/), licensed under a Creative Commons Attribution 4.0 Unported License. AD: Alzheimer’s disease; Aβ: amyloid-β; CTR1: copper transporter-1; Cu: copper; IL: interleukin; MT: metallothionein; NF-κB: nuclear factor-κB; ROS: reactive oxygen species; TNF-α: tumor necrosis factor-α.

Over the last 20 years, high free copper levels have been shown to be directly related to the pathogenesis of AD. High levels of free copper generate superoxide, hydrogen peroxide, and hydroxyl radicals that damage proteins, lipids, and DNA (Cheignon et al., 2018). AD and WD show common features in terms of pathogenesis, and zinc treatments for WD may also be applicable for AD. Zinc triggers intestinal MT production, which blocks copper absorption and increases copper excretion in the feces (Avan et al., 2022). Moreover, zinc-induced expression of MT proteins serves as a feedback mechanism for neuronal injury, inflammatory response, and oxidative stress (Aschner and West, 2005). In addition, Zn7-MT-3 can scavenge Cu^2+^ through its reduction to Cu^+^, and a metal swap between Zn7-MT-3 and Aβ peptides can eliminate ROS production and the subsequent cytotoxicity (Meloni et al., 2007, 2008). Likewise, in response to oxidative stress, the major human MT subtype, MT-2A, was secreted from astrocytes into the synaptic vicinity where Aβ peptides aggregated. Under physiological conditions, Zn7-MT-2A also exchanged Cu ions from Aβ to prevent ROS production and maintain metal homeostasis in neurons (Chung et al., 2010; **Figure 2**).

#### Metallothionein, copper, and Parkinson’s disease

**Figure 3:**
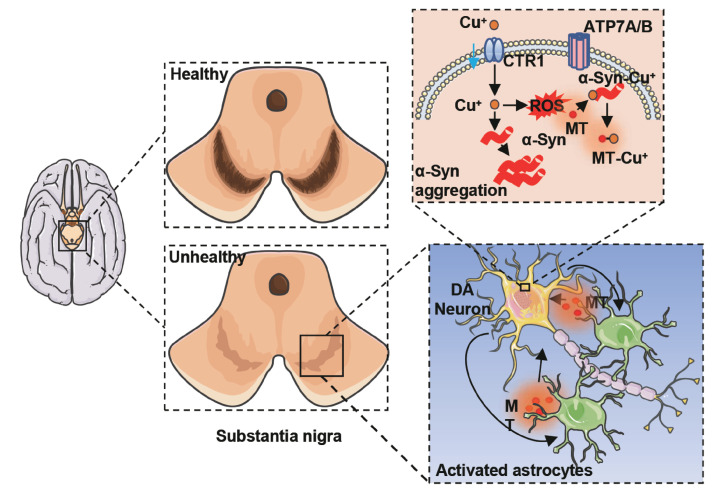
MT, copper, and PD. In PD, the adjustment of copper is regulated by CTR1 and ATP7A/B. The decreased level of CTR1 in DA neurons in the substantia nigra implies excessive aggregation of intracellular copper. Furthermore, excessive copper not only induced the aggregation of α-Syn but also produced a large amount of ROS, which caused DA neuronal injury. Moreover, injured DA neurons trigger the activation of astrocytes, which release MT to chelate copper to relieve metal overload in DA neurons. Created with Servier Medical Art (https://smart.servier.com/), licensed under a Creative Commons Attribution 4.0 Unported License. ATP7A/B: ATPase copper transporting alpha/beta; CTR1: copper transporter-1; Cu: copper; DA: dopamine; MT: metallothionein; PD: Parkinson’s disease; ROS: reactive oxygen species; α-Syn: α-synuclein.

In the brain, MT-1/2 expression occurred predominantly in astrocytes. Interestingly, neurons also synthesized MT-1/2, but the synthesis levels were several times less than those in astrocytes. MT-3 was expressed in neurons (Hidalgo et al., 1994; Masters et al., 1994). However, MT-3 localization is a topic of debate. Several studies have identified astrocytes as the primary source of MT-3. This difference may have occurred because neurons synthesized MT-3, and the MT-3 was then translocated to astrocytes (West et al., 2008; Howells et al., 2010). MT-copper has been recently shown to trigger the aggregation of α-synuclein proteins and generate ROS, further affecting the progression of PD (Adam et al., 2016). Moreover, MT knockout has been shown to block copper-induced reductions in DA levels in the midbrain and the frontal cortex (Petro et al., 2016). Taken together, these findings imply that MT chelates excess copper ions and acts as a powerful antioxidant (Okita et al., 2017). Similarly, additional studies indicated that in the presence of oxygen, α-synuclein-Cu^2+^ could oxidize DA to o-quinone, which involved a cyclic reaction between Cu^+^ and Cu^2+^. When MT-3 was added to the α-synuclein-Cu^2+^ complex, copper ions bound to α-synuclein were transferred to MT-3 and a series of subsequent oxidative damage indicators disappeared, demonstrating that MT-3 had a good affinity for copper ions (Meloni and Vašák, 2011). In addition, in patients with PD, postmortem brain tissue was found to show increased MT expression in the substantia nigra and cortical areas. This effect may be a compensatory mechanism of glial cells to alleviate oxidative stress (Michael et al., 2011; **Figure 3**).

#### Metallothionein, copper, and amyotrophic lateral sclerosis

**Figure 4:**
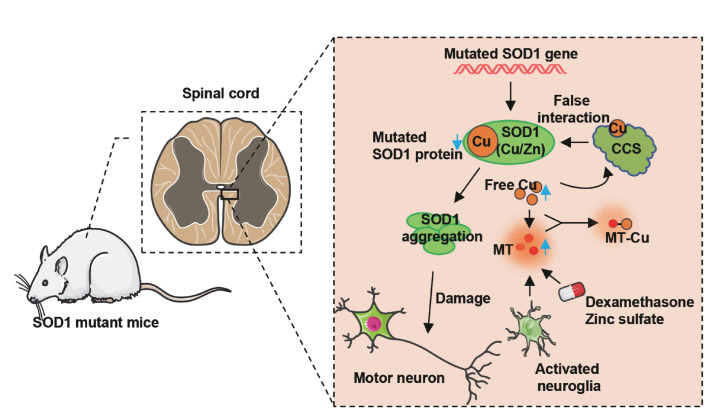
MT, copper, and ALS. In ALS, mutations in the SOD1 gene can result in a mutated SOD1 protein in the spinal cord, which accumulates in motor neurons and impairs copper-binding capacity, leading to an increase in free copper. Normally, CCS transports copper to Cu/Zn-SOD and activates SOD1 to produce antioxidant effects. During ALS, CCS interacts incorrectly with mutant SOD1, leading to the accumulation of unstable SOD1, which tends to become a pro-oxidant and thus exerts toxic effects on motor neurons. However, activated astrocytes and exogenous drugs (dexamethasone and zinc sulfate) can secrete MT to alleviate ALS progression. Created with Servier Medical Art (https://smart.servier.com/), licensed under a Creative Commons Attribution 4.0 Unported License. ALS: Amyotrophic lateral sclerosis; CCS: copper chaperone protein; Cu: copper; MT: metallothionein; SOD1: superoxide dismutase 1; Zn: zinc.

In transgenic SOD1^G93A^ mice, administration of dexamethasone induced endogenous MT expression to ameliorate the progression of ALS and rescue motor neurons. Surprisingly, the protective effects of dexamethasone were lost when endogenous MT was eliminated in SOD1^G93A^ mice, indicating that the efficacy of dexamethasone was dependent on endogenous MT (Tokuda et al., 2015). Similarly, in SOD1Tg mice, MT-1/2 expression increased along with copper levels in the spinal cord in an age-dependent manner, indicating that MT-1/2 attenuated this copper toxicity in the early stages of ALS (Tokuda et al., 2007). This adaptive response of MT to metal ion imbalance was evaluated in additional studies, where widespread activation of glial cells was found in SOD1^G93A^ mice at 3 months and increased gene expressions of MT-1 and MT-3 occurred at 4 months of age just prior to end-stage disease (Olsen et al., 2001). In overexpressing mSOD1^G93A^ transgenic mice, prolonged oral administration of zinc sulfate induced MT expression and reduced serum copper levels. However, the life span of the mSOD1^G93A^ mice decreased (Groeneveld et al., 2003; **Figure 4**).

#### Metallothionein, copper, and Wilson’s disease

**Figure 5:**
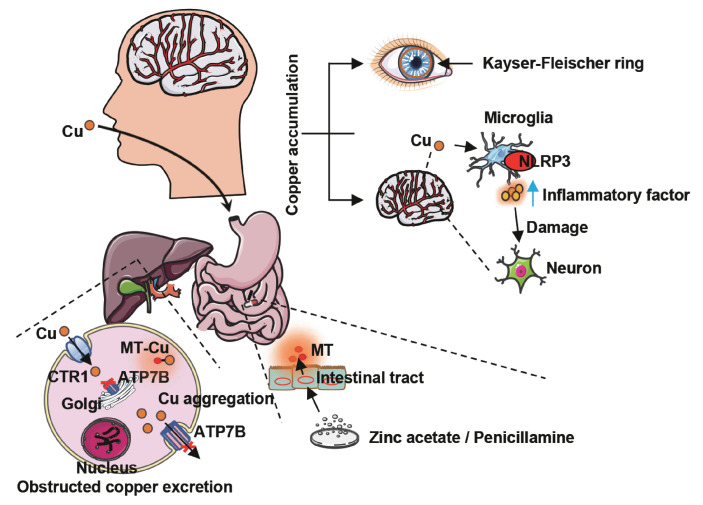
MT, copper, and WD. In WD, impaired ATP7B function affects copper efflux, leading to persistent copper accumulation in brain and eye tissues. Copper in the brain activated NLRP3 inflammasome signaling in microglia to produce proinflammatory factors that caused neuronal loss. In the eye, Kayser-Fleischer ring form when copper accumulates in the corneal region of the eye. Drugs used to treat WD disease are usually zinc acetate and penicillamine, which promote the secretion of MT by intestinal cells to chelate excess copper. Created with Servier Medical Art (https://smart.servier.com/), licensed under a Creative Commons Attribution 4.0 Unported License. ATP7B: ATPase copper transporting beta; CTR1: copper transporter-1; Cu: copper; MT: metallothionein; NLRP3: NOD-like receptor family pyrin domain containing 3; WD: Wilson’s disease.

In patients with WD, zinc salts are administered to stimulate MT synthesis in enterocytes. MT has a higher affinity for copper than zinc. Thus, MT chelates the dietary copper, limiting the transfer of copper into the portal circulation and decreasing the accumulation of copper ions in the liver (Brewer, 2009). Zinc acetate has been already approved by the U.S. Food and Drug Administration for maintenance therapy in adults and children with WD (Brewer, 2001). Penicillamine can also increase MT mRNA levels to treat WD (McArdle et al., 1990). Moreover, in the ATP7B^–/–^ mouse model, the abundance of MT-1/2 was regulated not only by tissue metal content (zinc and copper), but also by the redox state of the cell and serum growth factors, and variations in these regulators potentially weakened the abundance of MT, reducing the copper-consolidation capacity of the tissue (Muchenditsi et al., 2021; **Figure 5**).

#### Metallothionein, copper, and Huntington’s disease

In HD, copper has been shown to increase polyQ aggregation *in vivo* and *in vitro*. Overexpression of MT-3 in HeLa cells reduced polyQ aggregation and toxicity (Hands et al., 2010).

### Metallothionein, iron, and neurodegenerative diseases

#### Metallothionein, iron, and Alzheimer’s disease

**Figure 6:**
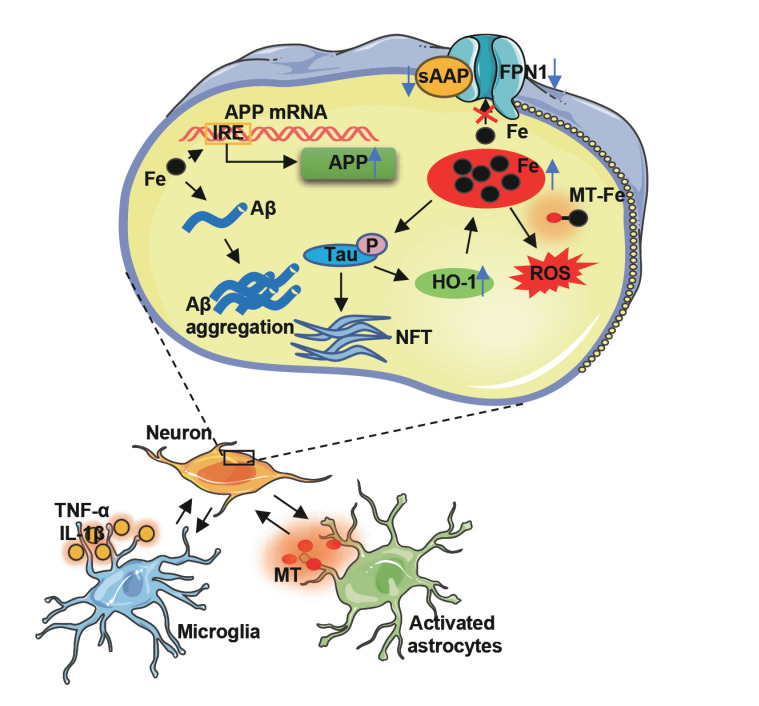
MT, iron, and AD. In AD, the nonamyloid pathway of neurons is inhibited, leading to a decrease in sAPP and further affecting the activity of FPN1, allowing iron to accumulate in neurons. On one hand, iron binds to the iron binding site on the *APP* gene and increases APP expression. On the other hand, iron promoted the phosphorylation of tau proteins to produce NFT, and phosphorylated tau increased the expression of HO-1 to induce ROS generation. Activated astrocytes in AD secrete MT to confer antineuroinflammatory and metallolytic effects. Created with Servier Medical Art (https://smart.servier.com/), licensed under a Creative Commons Attribution 4.0 Unported License. AD: Alzheimer’s disease; APP: amyloid precursor protein; Aβ: amyloid-β; Fe: iron; FPN1: ferroportin 1; HO-1: heme oxygenase-1; IL: interleukin; MT: metallothionein; NFT: neurofibrillary tangle; ROS: reactive oxygen species; sAPP: secreted form of APP; TNF-α: tumor necrosis factor-α.

Metal ions such as copper, iron, zinc, magnesium, and aluminum ions are involved in the progression of AD. Metal ions can affect neuronal metabolism and induce oxidative stress and promote Aβ deposition in the brain (Wang and Wang, 2017). This metal ion hypothesis highlights the role of abnormal redox cycling of metal ions in the aging process. Long-term excessive iron exposure can lead to neuronal synaptic damage and impaired cognitive function in AD. Therefore, the strategy of utilizing metal chelators to reduce excess iron in the brain of AD patients has gained much attention. The presence of large numbers of activated astrocytes and microglia along with elevated levels of inflammatory factors, such as TNF-α and IL-1β, has been demonstrated in AD (Kim et al., 2018). Meanwhile, activated astrocytes in AD secrete MT-1, which exerts anti-neuroinflammatory effects and plays a critical role in metal detoxification (Good and Vasák, 1986; Choo et al., 2018; **Figure 6**).

#### Metallothionein, iron, and Parkinson’s disease

**Figure 7:**
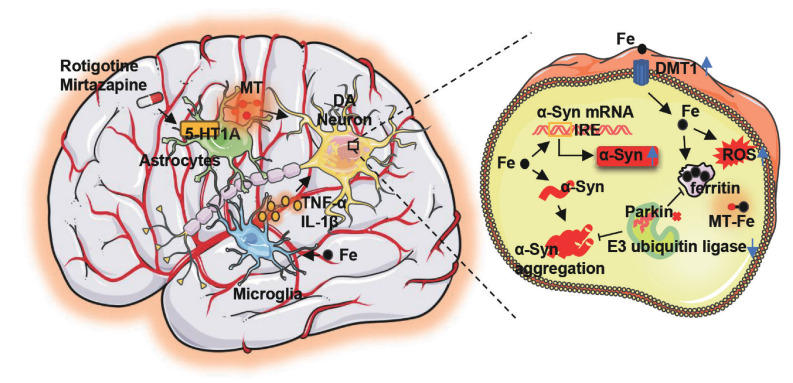
MT, iron, and PD. In PD, increased expression of DMT1 protein caused increased iron content in the DA neurons of the substantia nigra. The interaction of iron with the iron binding site on the *α-Syn* gene led to increased expression of the α-Syn protein. Moreover, iron also elicited the aggregation of α-Syn. Inactivation of the E3 ubiquitin ligase in PD inhibited the degradation of α-Syn and ferritin. However, rotigotine and mirtazapine protected DA neurons by promoting MT release via 5-HT1A receptors on astrocytes. Created with Servier Medical Art (https://smart.servier.com/), licensed under a Creative Commons Attribution 4.0 Unported License. 5-HT1A: 5-Hydroxytryptamine 1A; DA: dopamine; DMT1: divalent metal transporter 1; Fe: iron; MT: metallothionein; PD: Parkinson’s disease; α-Syn: α-synuclein.

Patients with PD show apparent iron aggregation in the substantia nigra. In the early stages of the disease, iron accumulation may be attributable to increased BBB permeability, the enhanced inflammatory state of the brain, and the high expression levels of divalent metal transporter 1 in DA neurons. Moreover, an *in vitro* study has revealed that the synthetic compound iron chelator could be utilized to protect neurons by inhibiting ferroptosis (Zeng et al., 2021). In addition, the natural endogenous chelator MT may play an essential role in the chelation of metallic irons. Rotigotine has been shown to inhibit 6-OHDA-induced dopaminergic neurotoxicity, and its mechanism may be related to the promotion of the expression of the antioxidant molecule MT-1/2 from astrocytes by stimulation of 5-hydroxytryptamine 1A (5-HT1A) receptors on astrocytes (Isooka et al., 2020). By the same mechanism of action, mirtazapine also upregulated MT expression via astrocyte 5-HT1A receptor activation to protect neurons against 6-OHDA-induced neurotoxicity (Kikuoka et al., 2020; **Figure 7**).

#### Metallothionein, iron, and Huntington’s disease

In HD, the disruption of brain iron homeostasis occurs before the onset of clinical symptoms. Iron accumulation may play an important role in the pathogenesis of HD. In HD, the mutant HTT protein shows increased expression of iron-regulated proteins such as iron regulatory protein 1, transferrin, and transferrin receptors, leading to iron accumulation (Niu et al., 2018). In addition, using single-nucleus RNA sequencing to identify cellular phenotypic changes in HD, gene expression of MT was shown to be upregulated in astrocytes (Al-Dalahmah et al., 2020). Similarly, MT expression was increased in astrocytes in the HD cingulate gyrus, and MT overexpression in astrocytes buffered glutamate and was neuroprotective against rotenone-induced neuronal death *in vitro* (Paryani et al., 2024). Interestingly, MT usually contains zinc or cadmium ions. However, iron ions could replace these ions (Good and Vasák, 1986). The existing studies have not clarified whether MT ameliorates HD by binding iron ions or eliminating the oxidative stress triggered by iron ions.

### Metallothionein, zinc, and neurodegenerative diseases

#### Metallothionein, zinc, and Alzheimer’s disease

**Figure 8:**
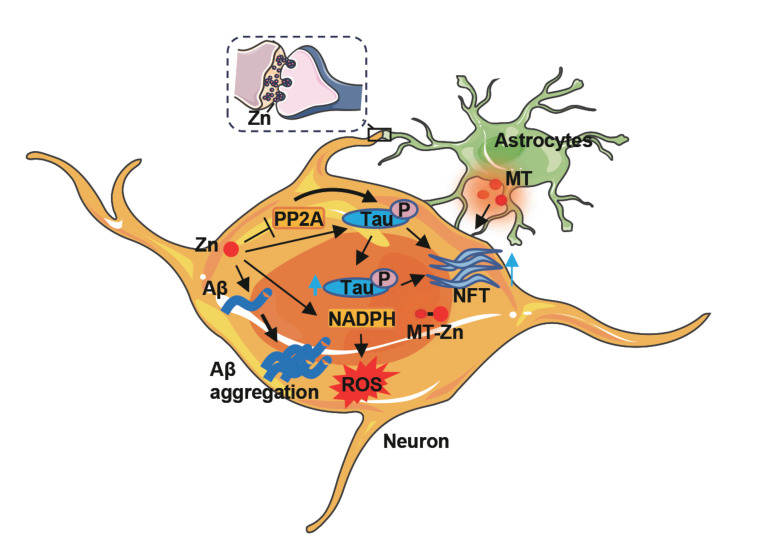
MT, zinc, and AD. In AD, increased levels of zinc ions cause Aβ aggregation. Moreover, zinc ions inhibited the ability of PP2A to phosphorylate tau proteins to aggregate into NFT. Moreover, zinc ions released by astrocytes in the synaptic gap activate NADPH enzymes to induce ROS production. However, astrocyte-released MT buffers the toxic effects of zinc ions. Created with Servier Medical Art (https://smart.servier.com/), licensed under a Creative Commons Attribution 4.0 Unported License. AD: Alzheimer’s disease; Aβ: amyloid-β; MT: metallothionein; NADPH: nicotinamide adenine nucleotide phosphate; NFT: neurofibrillary tangle; PP2A: protein phosphatase 2A; ROS: reactive oxygen species; Zn: zinc.

Aβ_1–42_ is the causative peptide of AD. In the presence of Aβ_1–42_, extracellular Zn^2+^ tends to form the (Zn^2+^-Aβ_1–42_) complex, inducing cognitive dysfunction in rats. Fortunately, MT can cushion Zn^2+^ when intracellular Zn^2+^ levels are dysregulated (Sato et al., 2021). Moreover, while aluminum is considered to be a major risk factor for AD, zinc is recognized as an important dietary microelement. Furthermore, zinc sulfate has been shown to reduce aluminum metal–induced lipid peroxidation and ROS levels by increasing MT expression in rat neurons (Singla and Dhawan, 2014; **Figure 8**).

#### Metallothionein, zinc, and Parkinson’s disease

**Figure 9:**
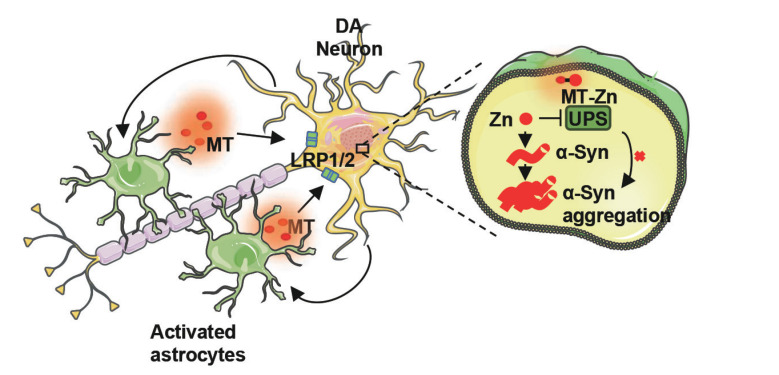
MT, zinc, and PD. In PD, increased levels of zinc ions led to the aggregation of α-Syn, whereas excess zinc ions inhibited the ubiquitin protease system (UPS), affecting the degradation of α-Syn. Increased α-Syn activated astrocytes to secrete MT, which entered DA neurons via the LRP1 and LRP2 receptors to chelate excess zinc ions to exert neuroprotective effects. Created with Servier Medical Art (https://smart.servier.com/), licensed under a Creative Commons Attribution 4.0 Unported License. DA: Dopamine; LRP1/2: low-density lipoprotein receptor-related protein 1/2; MT: metallothionein; PD: Parkinson’s disease; Zn: zinc; α-Syn: α-synuclein.

The pathology of PD is characterized by Lewy bodies that are formed by the aggregation of α-synuclein (Park et al., 2025; Sung and Chung, 2025). An imbalance in zinc ion levels is also a major pathogenetic factor in PD, with zinc ions promoting the accumulation of α-synuclein (Gao et al., 2022). Massive α-synuclein aggregation activates astrocytes to secrete MT-1/2, which protects neurons by low-density lipoprotein receptor family signaling via cell surface lipoprotein receptor-1 and megalin (lipoprotein receptor-2) (Pedersen et al., 2009; Okita et al., 2017). In addition, MT binds zinc with high affinity and acts as an intracellular zinc reservoir, thereby chelating excess zinc ions and regulating zinc release in case of zinc deficiency to maintain homeostasis of intracellular zinc levels (Baltaci et al., 2018). Under conditions of oxidative stress, modification of the glutathione redox balance has been shown to accelerate the release of metals from MT, and this disorder of metal metabolism has important implications for the progression of PD (Maret, 1995; **Figure 9**).

#### Metallothionein, zinc, and amyotrophic lateral sclerosis

The affinity of zinc was found to be reduced 30-fold in the mutant form of SOD1, while that of copper was unchanged. Meanwhile, zinc deficiency triggered the aggregation of mutant SOD1 (Sannigrahi et al., 2021). Not surprisingly, the elevated ROS levels triggered by the increase in free zinc levels may be a causative factor in the pathogenesis of ALS. Zinc regulatory proteins can be classified into two main types: MT and zinc transporter proteins. In the spinal cord of ALS patients, MT expression is upregulated (Sillevis Smitt et al., 1994). However, in SOD1 mutant mice, deletion of MTs accelerated ALS progression (Smith and Lee, 2007). These findings suggest that MT can scavenge excess levels of zinc and ROS.

### Mechanisms underlying the effects of metallothionein and copper, iron, and zinc ions in different neurodegenerative diseases

MTs, which are characterized by their Cys-rich composition and low molecular weight, play major regulatory roles through various pathways. A comprehensive investigation of the mechanisms involving MTs and metal ions in neurodegenerative diseases is expected to pave the way for novel preventive and therapeutic strategies for these conditions. In this article, we have reviewed the mechanisms by which MTs interact with copper, iron, and zinc ions in the context of different neurodegenerative diseases (**Additional Table 2**).

**Additional Table 2:** Mechanisms of MT and copper, iron and zinc ions in different neurodegenerative diseases

Neurodegenerative disease	Metal ion	Mechanism
Alzheimer's disease	Copper	MT regulates copper metabolism and reduces reactive oxygen species.
	Iron	MT-1 exerts antineuroinflammatory and metal detoxification effects.
	Zinc	MT buffers zinc dysregulation and reduces amyloid β-protein aggregation.
Parkinson's disease	Copper	MT chelates copper to reduce α-synuclein aggregation.
	Iron	MT protects neurons via 5-hydroxytryptamine receptor 1A receptor activation.
	Zinc	MT maintains zinc homeostasis through low-density lipoprotein receptor- related protein 1/2 signaling.
Amyotrophic lateral sclerosis	Copper	Dexamethasone enhances MT to improve copper ion homeostasis.
	Zinc	MT scavenges zinc and reactive oxygen species to slow disease progression.
Wilson's disease	Copper	Zinc salts and penicillamine promote MT synthesis to reduce copper accumulation.
Huntington's disease	Copper	MT-3 overexpression reduces polyglutamine aggregation and toxicity.
	Iron	Mutant proteins cause iron accumulation with MT upregulation in astrocytes.

MT: Metallothionein.

## Drugs Affecting Metallothionein in Neurodegenerative Diseases

8-OH-DPAT, a 5-HT1A agonist, can inhibit adenylate cyclase activity and reduce cyclic adenosine monophosphate production, which, in turn, regulates the balance of neurotransmitters such as 5-hydroxytryptamine, DA, norepinephrine, and acetylcholine (Assié et al., 1997; Polter and Li, 2010; Qin et al., 2014; Zhou et al., 2019). These monoamine transmitters play important roles in cognitive processes. Specifically, DA has been implicated in learning, memory, and reward mechanisms, while 5-hydroxytryptamine is associated with the regulation of mood and cognitive functions (Pillon et al., 2003; Ma et al., 2023). Neurotransmitter release and regulation are closely linked to metal ion homeostasis (Yoo et al., 2023). Interestingly, in a model of mercury-induced cognitive impairment, MT deficiency triggered alterations in monoamine transmitter levels that influenced cognitive function (Eddins et al., 2008). Neurotransmitter levels provide a stable chemical environment for normal functioning of MT, facilitating its involvement in nerve cell metabolism and protection. Collectively, these findings highlight the importance of clarifying the interactions of 5-HT1A agonists, MT, and monoamine transmitters in different physiological and pathological states to expand their applications in the treatment of neurodegenerative diseases.

Levodopa, an anti-PD drug, is converted to DA when it enters the brain and supplements DA (Huenchuguala and Segura-Aguilar, 2024; Shebl and Salama, 2025). However, the oxidative metabolism of DA generates large amounts of ROS, which cause oxidative stress in nerve cells (Chakrabarti and Bisaglia, 2023). Zinc-MT, as an important antioxidant, can protect nerve cells from oxidative damage during DA oxidative metabolism by regulating the levels of cellular zinc ions and enhancing the cellular antioxidant defense mechanism (Gauthier et al., 2008; Eibl et al., 2010). Dopamine agonists act directly on DA receptors to mimic DA function and maintain normal nerve cell activity and metabolism (Ferraiolo and Hermans, 2023). Moreover, astrocytes respond to DA by showing an increase in intracellular calcium ion concentration, which may modulate synaptic transmission and plasticity by releasing neuroactive substances (e.g., glutamate, gamma-aminobutyric acid, d-serine, and ATP) that affect MT binding to metal ions. These processes improve the effectiveness of MT in maintaining intracellular metal ion homeostasis and antioxidant capacity (Corkrum and Araque, 2021). As for monoamine oxidase B inhibitors, inhibiting DA degradation and increasing dopaminergic neurotransmission may enhance these mechanisms (Chaisanam and Wattanathorn, 2025).

The components of coffee (caffeic acid and chlorogenic acid) have been shown to upregulate the expression of the antioxidant molecule MT-1/2 in astrocytes in the striatum of mice and prevent rotenone-induced neurodegeneration of central dopaminergic nerves and peripheral enteric neurons, demonstrating that these components have a positive influence on the activation of MT in neurodegenerative diseases (Miyazaki et al., 2019).

Herbal monomers, including quercetin and Icariin, have been shown to activate the nuclear factor erythroid 2-related factor 2 (Nrf2) antioxidant signaling pathway, which plays an important role and is crucial in the context of neurodegenerative diseases (Zhang et al., 2019a; Cheng et al., 2024). Nrf2 is a key transcription factor responsible for regulating cellular antioxidant defense mechanisms (Ma, 2013). Nrf2 activates the expression of a range of antioxidant enzymes and proteins by binding to antioxidant response elements (AREs) (Guan et al., 2024; Kadam et al., 2024). In particular, Nrf2 can bind to the MT gene promoter and promote the transcription of MT, thereby increasing the expression level of MT (Zhou et al., 2017). Interestingly, the Nrf2 signaling pathway is over-activated and MT attenuates the oxidative stress of Nrf2 through feedback antioxidant regulation, both of which synergistically maintain the stability of the intracellular environment.

## Metallothionein Is a Hallmark of Neurodegenerative Diseases

For neurodegenerative diseases, the pathological process may commence at the cellular and molecular levels without manifesting overt clinical symptoms. MTs are responsive to alterations in the intracellular environment, and their expression levels may be altered during the early stages of disease, particularly in the presence of pathological factors such as oxidative stress and metal ion imbalances. Early diagnosis may be facilitated by detecting MT levels in the cerebrospinal fluid or blood. Cerebrospinal fluid, which is in direct contact with the CNS, offers a more precise indication of pathological alterations within the brain; however, its collection is relatively invasive (Vernau et al., 2008). Conversely, blood testing is more convenient and non-invasive, although blood levels of MT may be influenced by various systemic factors (Gellein et al., 2007; Shabb et al., 2017). Therefore, advancements in highly sensitive and specific assays, such as enzyme-linked immunosorbent assays and inductively coupled plasma mass spectrometry, are crucial for precise quantification of MT levels. Notably, the enzyme-linked immunosorbent assay is highly sensitive and can detect MT at the pg/mL level, but its results are affected by the quality of the antibody and sample treatment, while inductively coupled plasma mass spectrometry is more sensitive and can detect lower concentrations of MT, which mainly depends on the performance of the instrument and the sample pre-treatment. The enzyme-linked immunosorbent assay is dependent on the antibody, and antibodies with high specificity can reduce cross-reactions. On the other hand, while inductively coupled plasma mass spectrometry is highly specific, it requires pre-treatment to reduce matrix interference. MT detection methods currently suffer from a lack of standardization, which affects the comparability and reproducibility of the results, and establishment of consistent standards is essential. Future studies should optimize the performance of these assays and establish a standardized process to promote the clinical application of MT as a biomarker.

Several challenges are currently associated with the use of MT as an early diagnostic marker. First, the physiological range of MT can vary significantly among individuals, and is influenced by factors such as age, sex, and lifestyle. Second, alterations in MT patterns may overlap among various neurodegenerative diseases, complicating their use as specific diagnostic indicators. Consequently, additional research is essential to delineate the specific changes in MT associated with distinct disease states. As neurodegenerative diseases progress, MT expression levels may undergo additional alterations.

MT shows great potential for the differential diagnosis of neurodegenerative diseases. The unique expression patterns of MT isoforms in different diseases allow the distinction of AD, PD, ALS, WD, and HD. For example, in AD, the combination of decreased MT-3 levels and increased MT-1/2 levels represents a unique signature that distinguishes AD from other diseases like PD. In PD, MT-1/2 levels rise mainly due to stress in dopaminergic neurons. In ALS, the interaction between MT expression and copper homeostasis, especially in cases with SOD1 mutations, can serve as a diagnostic indicator to distinguish ALS from other motor neuron diseases. WD is characterized by increased MT-1/2 levels in the serum and liver, which helps distinguish it from other copper-related diseases. Similarly, in HD, the upregulation of MT in astrocytes and its interaction with polyQ aggregates provide specific biomarkers.

Combining MT with the dynamic response of disease-specific metal ion imbalances can enhance its application in differential diagnosis. For instance, the interaction of MT with copper and zinc ions in AD can be distinguished from copper detoxification in WD. Similarly, the iron and copper chelation of MT in PD can be distinguished from the zinc-related pathology in ALS. These specific interaction profiles of MT with metals can be used to develop diagnostic algorithms that combine metal ion levels with MT expression.

Moreover, combining MT levels with clinical symptoms and genetic information can improve diagnostic accuracy. In early-onset AD with a family history, the combination of low MT-3 levels with cognitive decline and positive amyloid protein biomarkers can solidify the diagnosis. In contrast, in PD, the elevation of MT-1/2 levels along with motor symptoms and dopamine deficiency can help distinguish it from secondary parkinsonism.

Developing MT-based diagnostic methods requires multicenter validation studies to establish cutoff values and sensitivity/specificity curves for each disease. These methods should include MT isoforms and other disease-specific biomarkers, such as phosphorylated tau protein for AD, α-synuclein for PD, and TDP-43 for ALS. Machine learning algorithms can further optimize these methods by identifying complex biomarker interactions that are not evident through traditional statistical methods.

In summary, MT-based diagnostic methods can significantly enhance the differential diagnosis of neurodegenerative diseases by providing disease-specific biomarkers. However, ongoing research is required to overcome the current challenges and establish standardized protocols for widespread clinical application.

Pharmacological agents used for treating neurodegenerative diseases may affect the expression of MT. For example, some antioxidant drugs can indirectly modulate MT levels by mitigating oxidative stress. Effective pharmacotherapy may normalize or favorably alter the trajectory of MT expression. Monitoring these changes can provide timely insights into the efficacy of treatment, facilitating necessary adjustments to therapeutic regimens and allowing the identification of more potent therapeutic agents. In clinical drug trials, monitoring biomarkers such as MT could serve as a valuable tool for assessing therapeutic outcomes (Sharma et al., 2013).

## Therapeutic Agents Targeting Metallothionein for Neurodegenerative Diseases: Clinical or Preclinical Studies and Limitations

Currently, no drug that specifically employs MT as the principal component for the treatment of neurodegenerative diseases has been approved by the U.S. Food and Drug Administration, China Food and Drug Administration, or World Trade Organization (WTO) clinical trial registries. In the realm of drug research, MT-based therapies for neurodegenerative disorders have faced numerous obstacles. The pathogenesis of neurodegenerative diseases is highly intricate, involving protein misfolding, neuroinflammation, oxidative stress, and other contributing factors. Although MT exhibits metal-chelating and antioxidant properties, its precise mechanisms of action within this complex disease pathology remain to be elucidated, complicating the identification of target sites and the design of effective drugs. Furthermore, accurately replicating the human physiological state and disease micro-environment in animal models or *in vitro* experiments remains challenging, resulting in discrepancies between preclinical study outcomes and actual clinical applications.

Therapy strategies targeting MT have also faced numerous challenges. First, determining the optimal mode and dosage of administration is exceedingly complex. The presence of the BBB represents a major impediment to efficient delivery of MT to the brain. Additionally, the absorption, distribution, metabolism, and excretion of the drug show considerable interindividual variations, complicating the precise determination of appropriate dosages. Second, the long-term safety of MT requires thorough evaluation. Given its role as a metal chelator, excessive chelation of metal ions could potentially disrupt the homeostasis of metal ions in the body, leading to a series of adverse reactions. Third, the clinical translation of MT therapy for neurodegenerative diseases faces substantial challenges, particularly due to the prolonged nature of these diseases and the gradual progression of their symptoms. To accurately evaluate the efficacy and safety of pharmaceutical interventions, long-term, large-scale clinical trials are required, which inherently necessitate substantial investments of time, financial resources, and human capital. Moreover, even upon the successful development of an efficacious drug, market dynamics and cost considerations remain critical. Patients with neurodegenerative disorders often require prolonged treatment regimens, necessitating a balance between affordability of the drug for both patients and health insurance systems as well as the need to ensure a reasonable return on investment after accounting for production and research costs.

## Limitations

The search strategy was limited to the PubMed database, which may have excluded relevant studies from other sources or non-English publications. The studies spanned from 1957 to 2025, and earlier studies may have lacked the methodological rigor of recent studies, introducing potential heterogeneity. The inclusion criteria focused on direct links between MT and neurodegenerative diseases, possibly excluding indirectly related studies. The analysis was qualitative, limiting precise effect estimates. Lastly, variability in experimental designs and reporting across studies may have affected the consistency of the results.

## Conclusion and Perspectives

Essential metals such as copper, iron, and zinc are critical for maintaining the physiological homeostasis of the human body, although at relatively low concentrations. These metals are also widely present in metal regulatory proteins as metal cofactors, which play crucial roles in the regulation of metal ion homeostasis. The metal ion hypothesis has become a hot topic, and has led to the identification of more forms of programmed cell death, such as cuproptosis, ferroptosis, and zincosis, which are highly associated with the pathogenesis of neurodegenerative diseases.

In addition, a common feature of most neurodegenerative diseases is the deposition of pathogenic proteins, including Aβ peptides and tau, α-synuclein, TDP-43, and HTT. Under pathological conditions, metal ion homeostasis is disrupted, and these pathogenic proteins are susceptible to binding to metal ions, which triggers a series of protein aggregation and ROS cascade reactions that further advance the pathology of neurodegenerative diseases.

The main reasons for excessive levels of metal ions in the human body are as follows: (1) Environmental pollution: Industrial and mining activities may lead to heavy metal contamination. These metals enter the ocean through rivers, affecting water quality, and may accumulate in the human body through the food chain. (2) Occupational exposure: In certain industries, such as electrical, electronics, battery manufacturing, and welding and cutting industries, workers may be at risk of excessive intake due to long-term contact with substances containing heavy metals. (3) Metal implants: Medical devices, such as metal implants in the body, may slowly release metal ions due to wear and corrosion, resulting in an excess of metal ions in the body. (4) Drug misuse: The abuse of certain medications, such as traditional Chinese and Western medicines containing heavy metal components, may also lead to excessive levels of heavy metals in the human body, and can cause harm to the body. (5) Crop and aquatic product absorption: Several crops and aquatic products absorb heavy metal elements from the soil and water bodies during their growth process, which can also lead to excessive intake of heavy metals by the human body.

Apart from MT-based approaches, various approaches exist to reduce the levels of metal ions within the body. Some examples of these approaches are as follows: (1) Increased excretion and metabolism by enhancing the body’s metabolic rate, such as by drinking more water and maintaining proper exercise, promotes metabolism in the liver or skin, helping expel heavy metals. (2) Diet adjustment by consuming more cruciferous vegetables, such as cauliflower and broccoli, can help in the excretion of heavy metals. (3) Nutritional supplementation using antioxidants, such as vitamin C, vitamin E, β-carotene, and selenium, can help reduce oxidative stress and decrease the damage caused by metal ions. These approaches can serve as supplementary methods for prevention and treatment of excessive metal ions in the body, thereby reducing the impact of heavy metals on health.

Currently, metal ion chelators have been recognized as potential therapeutic agents for diseases involving metal ion imbalances. However, synthetic metal ion chelators tend to have greater adverse reactions and higher production costs, limiting their utility in the treatment of neurodegenerative diseases. Conversely, the natural metal ion chelator MT not only shows excellent therapeutic effects, but also has fewer side effects. MT shows substantial therapeutic potential in neurodegenerative diseases, and future studies need to explore the activation mechanisms and therapeutic potential of MT. To this end, identification of alternatives from natural compounds that can activate endogenous MT to alleviate the progression of neurodegenerative diseases is essential.

## Additional files:

***Additional Table 1:*** *Characterization of Alzheimer’s disease, Parkinson’s disease, amyotrophic lateral sclerosis, Wilson’s disease, and Huntington’s disease.*

***Additional Table 2:*** *Mechanisms of MT and copper, iron and zinc ions in different neurodegenerative diseases.*

## Data Availability

*All relevant data are within the paper and its Additional files*.
